# Prevalence of anaemia and associated factors among infants under 6 months in rural China

**DOI:** 10.1017/S1368980022001616

**Published:** 2023-03

**Authors:** Yefan Du, Anne Durstenfeld, Sarah-Eve Dill, Qingzhi Wang, Huan Zhou, Hao Xue, Saraswati Kache, Alexis Medina, Scott Rozelle

**Affiliations:** 1 Department of Health Behavior and Social Science, West China School of Public Health and West China Fourth Hospital, Sichuan University, No. 16 Section 3 South Renmin Road, Chengdu, Sichuan 610041, People’s Republic of China; 2 Department of Pediatrics, Division of Pediatric Critical Care Medicine, Stanford University, Palo Alto, CA, USA; 3 Rural Education Action Program, Freeman Spogli Institute for International Studies, Stanford University, Palo Alto, CA, USA

**Keywords:** Infants under 6 months, Anaemia, Low birth weight, Caesarean section, Rural western China

## Abstract

**Objective:**

To examine Hb level and anaemia status among infants under 6 months of age in rural China.

**Design:**

A cross-sectional survey collected data among infants under 6 months and their primary caregivers in Sichuan, China. Anaemia was defined using both the WHO and China Pediatrics Association thresholds. Multivariable linear regression was used to identify relevant factors among two age groups (<4 months; 4–5 months).

**Setting:**

Eighty townships were selected in Sichuan, China from November to December 2019.

**Participants:**

Nine hundred and forty-two infants under 6 months, while Hb level was tested for 577 infants.

**Results:**

The overall mean (±sd) Hb level was 106·03 (± 12·04) g/l. About 62·6 % (95 % CI 58·5, 66·6) of sample infants were anaemic using the WHO threshold, and 20·5 % (95 % CI 17·3, 24·1) were anaemic using the China Pediatrics Association thresholds. Anaemia rates rose with increasing age in months. Multivariable linear regressions revealed that lower Hb levels were significantly associated with lower birth weight (<4 months: *β* = 4·14, 95 % CI 0·19, 8·08; 4–5 months: *β* = 6·60, 95 % CI 2·94, 10·27) and delivery by caesarean section (<4 months: *β* = −4·64, 95 % CI −7·79, −1·49; 4–5 months: *β* = −4·58, 95 % CI −7·45, −1·71).

**Conclusion:**

A large share of infants under 6 months in rural western China are anaemic. Infants with low birth weight and caesarean delivered should be prioritised for anaemia testing. Future studies should move the point of focus forward to at least 4 months of age and examine the link between caesarean section and anaemia to promote health and development in infancy.

Iron deficiency anaemia disproportionately affects children under 5 years of age in low- and middle-income countries (LMIC), with potentially irreversible effects on cognitive function^(1,2)^. The WHO estimates that 43 % of children under 5 years of age are anaemic worldwide, and that 42 % of childhood anaemia is due to iron deficiency^(3)^. Iron deficiency anaemia during infancy and early childhood can lead to persistent deficits in brain function and lead to the loss of fitness and work capacity in the future life^(4,5)^.

While the majority of research on anaemia in young children has focused on the 6–59-month age group, little has been published on infants under 6 months. Compounding this problem is the lack of a global standard for anaemia in infants under 6 months, since the WHO threshold begins at 6 months of age^(3)^.

However, based on the high prevalence of anaemia in children aged 6–59 months in LMIC, many infants may have iron deficiency and undetected iron deficiency-based anaemia even before 6 months of age^(6,7)^. The WHO considers infants under 6 months to be protected from iron deficiency through breast-feeding^(3)^. However, for this assumption to be accurate, multiple factors are presumed: that the infant was born at full term with normal birth weight; had delayed umbilical cord clamping assuring adequate iron stores for the first 4–6 months of life and was provided with sufficient dietary iron through exclusive breast-feeding through 6 months^(3,8)^. Infants in LMIC, however, often do not fulfil all of these assumptions. Weaning, or the transition from exclusive breast-feeding to the addition of complementary foods, often occurs around 4–6 months of age when an infant’s iron stores are being depleted^(3)^. UNICEF data indicate that a third of 4–5-month-olds have already been fed complementary foods, which are frequently of poor nutritional value and low in iron, placing infants at high risk for the development of iron deficiency and anaemia during this period^(3,9)^.

The few empirical studies evaluating anaemia among infants under 6 months of age have reported a high prevalence of anaemia. Using the WHO threshold for anaemia, studies in infants under 6 months reported anaemia prevalence of 2·7 % in Taiwan, 50 % in South Africa, 71 % in Indonesia and 77 % in Peru^(10–13)^. One prior study in rural China used a threshold from the China Pediatrics Association with <90 g/l for under 4 months and <100 g/l for 4–5 months and reported a prevalence of over 20 % in infants under 6 months^(14,15)^. There is a need for more in-depth research to examine the Hb concentrations of infants under 6 months in multiple settings, particularly in rural areas and LMIC where the prevalence of early childhood anaemia is highest^(2)^.

Many of the same factors associated with anaemia in older infants may also predict anaemia in infants under 6 months. These include pregnancy and birth-related factors such as maternal anaemia, prematurity and low birth weight^(8)^. A previous study of infants 3–5 months old in Indonesia found that the associated factors for anaemia were maternal anaemia, low birth weight and child stunting^(12)^. In contrast, feeding practices such as micronutrient supplementation have been found to be protective against early childhood anaemia^(16–18)^, and the WHO recommends iron supplementation starting as early as 2 months for infants with low birth weight^(3)^.

In China, despite several health care projects to prevent anaemia have been launched in poor areas in recent decades, such as Ying Yang Bao (a free, government-distributed micronutrient powder for infants that contains iron), the prevalence of iron deficiency anaemia among young children in rural areas is still at a high level, with many of the same associated factors identified in the global literature^(19,20)^. Studies have reported the prevalence of anaemia among infants and toddlers aged 6–36 months to be between 35 and 53 % in rural areas, compared with 11–28 % in urban areas^(15,21–23)^. The highest prevalence of anaemia in rural China has been found among children 6–12 months of age in less-developed western regions with risk factors including maternal anaemia, prematurity and low birth weight^(24,25)^. Only one study examined the prevalence of anaemia among infants in rural China including those under 6 months. Results have shown than low birth weight, having siblings, low maternal education, low family income and inappropriate complementary food introduction were associated factors for anaemia^(15)^. However, this study did not exclusively focus on infants under 6 months and did not identify age-specific associated factors, making it difficult to determine whether these associated factors are accurate for infants under 6 months specifically.

To fill these gaps in the literature, our study reports the prevalence of anaemia and factors associated with lower Hb among infants under 6 months of age in a poverty-stricken rural area in western China. To do so, we pursue three specific objectives. First, we describe the prevalence of anaemia among the full sample and by age group (< 4 months and 4–5 months). Second, we chart the prevalence of anaemia by age in months. Third, we identify perinatal characteristics and feeding characteristics associated with lower Hb concentrations among infants under 6 months of age, both for the full sample and by age group. Based on previous studies in the literature, we hypothesise that anaemia prevalence will be relatively high among infants under 6 months in rural western China, be correlated with increasing age in months and be associated with prematurity, birth weight and feeding practices.

## Methods

### Study population and sampling

The data presented in this study were collected from November to December 2019 in four nationally designated rural poverty countries in a prefecture of Sichuan Province. Sichuan is located in the interior of southwestern China. The province is considered to be middle income, ranking sixth out of China’s thirty-one provinces in terms of GDP per capita. Despite its middle-income status, this province contains large shares of low-income populations, particularly in rural areas. Of the 132 counties in Sichuan Province, sixty-six are nationally designated poverty counties, including the four counties in this study. Additionally, the majority of rural residents in the four study counties are Han ethnicity, which accounts for over 90 % of China’s population (National Bureau of Statistics of China, 2011).

**Fig. 1:**
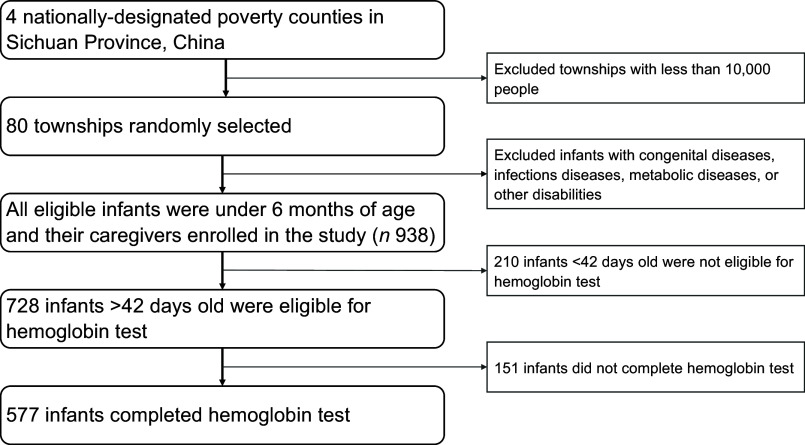
Flow chart of sample selection in Sichuan, China from November to December 2019

The study team followed a multistep protocol to select the study sample (Fig. 1). The first step selected townships within the four sample counties. A canvas survey was conducted between March and May 2019 to yield a list of townships. Urban townships and townships with less than 10 000 people were excluded from the sampling frame. From the remaining list of townships, eighty townships were randomly selected for inclusion in the final sample.

In the second step, the research team selected sample infants and households for the study. In each sample township, the study team obtained a list of all infants in the target age range (under 6 months) from the local township health centre. Infants with serious congenital diseases, infectious diseases (such as malaria, HIV and celiac disease), genetic metabolic diseases or other disabilities were excluded from this study. All remaining infants and their caregivers were enrolled in the study, totalling 728 infants. Of these, 210 infants under 42 d of age were not eligible for Hb testing^(26)^ and 151 infants did not complete the Hb test due to the caregiver’s refusal to cooperate or the failure of blood test due to infants’ own poor condition at the time (such as extreme wasting or low weight). The final analytical sample in this study therefore included 577 infants and their caregivers in eighty townships. A balance test comparing the characteristics of the 577 infants who completed the Hb test and the 151 infants who did not completed, and the result showed that the two groups were balanced in terms of demographic characteristics, perinatal characteristics and feeding behaviours (see online supplementary, Appendix Table 1).

### Data collection

Data were collected through a survey administered by trained enumerators in the home of each sample infant. Enumerators first identified each infant’s primary caregiver by asking which family member was most responsible for the infant’s health and daily care. The enumerators then conducted structured survey interviews with the primary caregiver.

The survey collected four blocks of data. The first survey block collected data on the Hb concentrations of sample infants. Hb tests were conducted by trained professionals using HemoCue 201+ (HemoCue AB). HemoCue 201+ is a portable haemoglobinometer that provides a point-of-care testing from a capillary sample, using proprietary cuvettes to facilitate calibration^(27)^. Testers preferentially selected the middle or ring finger of the infant’s left hand for capillary blood sampling and discarded the first drop of blood for accuracy. During the field, the blood collection device is calibrated daily to minimise the Hb measurement error. Since Hb concentrations increase by 4 % for every 1000 m of altitude above sea level^(28)^, all Hb measurements in this study were standardised by altitude for statistical analysis.

The second survey block collected data on the perinatal characteristics of sample infants. These include birth weight (in kg), gestational age at birth (in weeks), delivery method (vaginal birth or caesarean section (C-section)), whether infants had illnesses symptoms in the last 2 weeks (fever, cold, diarrhoea, pathological vomiting or blood in the stool), whether the mother experienced gestational anaemia and whether the mother had previously experienced a miscarriage/abortion. We obtained the infant’s age, gestational age, birth weight and birth length from the child’s birth certificate.

The third survey block assessed infant feeding behaviours. Each infant’s primary caregiver was administered a dietary questionnaire of all foods consumed by the infant in the previous 24 h, derived from the WHO *Indicators for Infant and Child Feeding* ^(29)^. The dietary questionnaire was then used to determine whether the infant received a diet of exclusive breast-feeding, mixed breast-feeding or non-breast-feeding. Following the WHO indicators, exclusive breast-feeding is defined as infants consuming only breast milk with no other foods or liquids (including water). Mixed breast-feeding is defined as infants receiving both breast milk and other foods or liquids, including water, non-human milk and formula. Non-breast-feeding is defined as infants receiving no breast milk and instead consuming other foods or liquids, including non-human milk and formula. We also collected information on whether the infant received iron supplementation, which was defined as consuming iron-fortified foods or receiving micronutrient supplements such as Ying Yang Bao in these regions.

The final survey block collected information on the socio-demographic characteristics of infants and households. Infant characteristics included age, gender and birth order. Household characteristics included mother’s age, education level and employment status, as well as the total self-reported household income (from all income sources by all members of the household) in the previous year. To assess caregiver educational level, caregivers were asked to report the highest level of schooling they had achieved (including ‘no formal education’, ‘did not finish primary school’, ‘primary school’, ‘junior school’, ‘high school’ and ‘undergraduate or higher level’). To increase the robustness of our results, we transformed the responses into a binary variable where 1 = completed 9 years education and 0 = less than 9 years education. To assess caregiver employment status, caregivers were asked to report their current occupations. Because nearly three quarters of caregivers reported that they were not working (i.e. were full-time caregivers), we condensed responses we transformed the responses into a binary variable where 1 = currently employed and 0 = not employed.

### Ethical approval

All participating caregivers gave their informed written consent for their own and their infants’ participation in the study. Infants found to have severe anaemia were referred to the local hospital for treatment.

### Statistical analysis

Our statistical analysis is comprised of three parts. First, we describe the prevalence of anaemia among the sample. Because there is no internationally established cut-off for anaemia among infants under 6 months, we use two diagnostic thresholds to define anaemia. The first threshold is the WHO threshold for children aged 6–59 months^(3)^. This threshold has been used in multiple studies of anaemia among infants under 6 months internationally^(11–13)^. Importantly, however, that cut-off is not established as a definitive threshold for infants under 6 months. We therefore use an additional threshold proposed by the China Pediatrics Association for infants under 6 months^(14)^. Using both thresholds, we report the prevalence of anaemia for both the full sample and the two age groups defined by China Pediatrics Association (< 4 months and 4–5 months) and conduct *χ* ^2^ tests to compare the prevalence of anaemia between the two age groups. In the second part of our statistical analysis, we chart the prevalence of anaemia by age in months, using both the WHO threshold and the China Pediatrics Association threshold to define anaemia.

In the third part of our analysis, we conduct a multivariable linear regression analysis to identify perinatal characteristics and feeding characteristics associated with Hb concentrations among the sample. We did not examine correlations to anaemia directly, since the definition of anaemia among infants under 6 months of age is not well established. The analysis controls for the socio-demographic characteristics of infants and households, including infant age, gender and birth order; maternal age, education level and employment status; and annual household income.

All statistical analyses were performed using STATA 15.0. Standard errors were clustered at the township level to control for any possible intra-township correlation. *P* values below 0·05 were considered statistically significant.

## Results

### Sample characteristics

Table 1 reports the summary statistics for the overall sample as well as separately for each age group (< 4 months and 4–5 months). For demographic characteristics of infants and caregivers, the mean (±sd) age of infants in the sample was 121·09 (± 43·59) d, with 266 infants under 4 months of age (42–119 d) and 311 infants aged 4–5 months (120–179 d). Female infants made up 44·0 % of our cohort, and approximately 43·2 % of infants were the firstborn child. The mean (±sd) maternal age was 28·19 (± 4·97) years. The majority of mothers (60·1 %) had completed 9 or more years of education, and 24·1 % of mothers were employed at the time of the survey. The mean (±sd) reported income of sample households in the previous year was 71 100 (± 57 000) RMB, or about $11 013 USD. Socio-demographic characteristics were not significantly different between the older and younger age groups.

**Table 1:** Basic characteristics of sample infants and households in Sichuan Province (*n* 577)

	Full sample (*n* 577) (1)		< 4 months (*n* 266) (2)		4–5 months (*n* 311) (3)		Difference* (2)–(3)
	*n* or mean	% or sd	*n* or mean	% or sd	*n* or mean	% or sd	
Socio-demographic characteristics							
Infant age (days)	121·09	43·59	81·88	22·23	154·63	25·75	<0·001
Gender							0·854
Male	323	55·98	150	56·39	173	55·63	
Female	254	44·02	116	43·61	138	44·37	
Birth order							0·224
Firstborn	249	43·15	122	45·86	127	40·84	
Second or higher	328	56·85	144	54·14	184	59·16	
Maternal age (years)	28·19	4·97	28·26	5·14	28·13	4·82	0·772
Maternal education							0·397
≤9 years	230	39·86	111	41·73	119	38·26	
>9 years	347	60·14	155	58·27	192	61·74	
Mother is employed							0·857
No	438	75·91	201	75·56	237	76·21	
Yes	139	24·09	65	24·44	74	23·79	
Annual household income (¥, 10^4^)	7·11	5·70	6·95	5·25	7·26	6·07	0·411
Perinatal characteristics							
Gestational age (weeks)	38·83	1·47	38·88	1·42	38·79	1·52	0·437
Birth weight (kg)	3·23	0·45	3·22	0·42	3·25	0·47	0·482
Delivery method							0·894
Vaginal birth	249	43·15	114	42·86	135	43·41	
Caesarean section	328	56·85	152	57·14	176	56·59	
Illness in the last 2 weeks							0·108
No	322	55·81	158	59·40	164	52·73	
Yes	255	44·19	108	40·60	147	47·27	
Gestational anaemia							0·436
No	310	56·57	142	54·83	168	58·13	
Yes	238	43·43	117	45·17	121	41·87	
Mother had previous miscarriage/abortion							0·698
No	310	56·47	144	55·60	166	57·24	
Yes	239	43·53	115	44·40	124	42·76	
Infant feeding behaviours							
Breast-feeding status							<0·001
Exclusive breast-feeding	177	30·68	127	47·74	50	16·08	
Mixed breast-feeding	372	64·47	127	47·74	245	78·78	
Non-breast-feeding	28	4·85	12	4·51	16	5·14	
Iron supplementation							0·002
No	474	82·15	233	87·59	241	77·49	
Yes	103	17·85	33	12·41	70	22·51	

* The difference between the two columns is represented by *P* values.

In terms of perinatal characteristics of the sample, the mean (±sd) gestational age was 38·83 (± 1·47) weeks, and the mean (±sd) birth weight was 3·23 (± 0·45) kg. The majority of infants were born by C-section (56·9 %). About 44 % of infants had symptoms of illness in the past 2 weeks. Almost half of mothers (43·4 %) reported having gestational anaemia, and 43·5 % of mothers reported having previously not completed a pregnancy because of miscarriage or abortion. The perinatal characteristics for infants under 4 months of age were not significantly different from those for infants 4–5 months of age.

In terms of infant feeding behaviours, the proportion of exclusively breastfed infants was 30·7 % overall, and this rate declined significantly by infant age, falling from 47·7 % among infants under 4 months to 16·1 % among infants aged 4–5 months (*P* < 0·001). About 64·5 % of infants in the full sample received mixed breast-feeding (47·7 % of infants < 4 months and 78·8 % of infants aged 4–5 months), and only 4·9 % of infants were not receiving any breast milk at the time of the survey (4·5 % of infants < 4 months and 5·1 % of infants aged 4–5 months). Among mixed breast-feeding and non-breast-feeding infants, 55·5 % consumed formula, 25·5 % consumed complementary foods and 11·8 % consumed animal milk the day before the survey. And 17·9 % infants in the full sample were receiving iron supplementation at the time of the survey (12·4 % of infants < 4 months and 22·5 % of infants aged 4–5 months; *P* = 0·002).

### Prevalence of anaemia

Table 2 reports Hb concentrations and anaemia rates for the sample. The mean (±sd) Hb level for the full sample was 106·03 (± 12·04) g/l, with a mean (±sd) of 105·13 (± 11·89) g/l among infants under 4 months and 106·80 (± 12·14) g/l among infant 4–5 months. Using the WHO threshold for anaemia (<110 g/l), the results show an anaemia prevalence of 62·6 % (95 % CI 58·5, 66·6) among the full sample, 65·4 % (95 % CI 59·3, 71·1) among infants under 4 months and 60·1 % (95 % CI 54·5, 65·6) among infants 4–5 months, with no significant difference in anaemia prevalence between the two age groups. When the China Pediatrics Association threshold for anaemia (<90 g/l for < 4 months and <100 g/l for 4–5 months) is used, the results show an overall anaemia prevalence of 20·5 % (95 % CI 17·3, 24·1), with a significantly higher prevalence of anaemia among infants aged 4–5 months (27·3 %, 95 % CI 22·5, 32·7) compared with infants under 4 months (12·4 %, 95 % CI 8·8, 17·1; *P* < 0·001).

**Table 2:** Hb concentrations and anaemia prevalence among sample infants in Sichuan Province (*n* 577)

	Full sample (*n* 577) (1)		< 4months (*n* 266) (2)		4–5 months (*n* 311) (3)		Difference* (2)–(3)
	*n* or mean	% or sd	*n or* mean	% or sd	*n* or mean	% or sd	
Hb, g/l	106·03	12·04	105·13	11·89	106·80	12·14	0·097
Anaemia status using WHO threshold†,‡							
Anaemic	361	62·56	174	65·41	187	60·13	0·191
Not anaemic	216	37·44	92	34·59	124	39·87	
Anaemia status using China Pediatrics Association threshold‡,§							
Anaemic	118	20·45	33	12·41	85	27·33	<0·001
Not anaemic	459	79·55	233	87·59	226	72·67	

* The difference between the two columns is represented by *P* values.

† To date, there is no universally established diagnostic threshold for anaemia among infants under 6 months. In this study, we therefore use two thresholds to define anaemia.

‡ The WHO has established a threshold of Hb <110 g/l for anaemia among infants over 6 months of age^(3)^.

§ The China Pediatrics Association threshold for anaemia among infants under 6 months is Hb <90 g/l for infants under 4 months and <100 g/l for infants aged 4–5 months^(14)^.

**Fig. 2:**
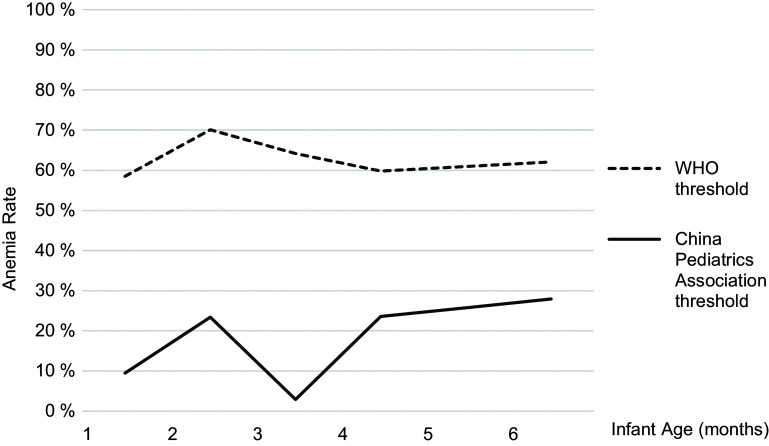
Anaemia distribution among sample infants in Sichuan Province using different diagnostic criteria (*n* 577). Because there is no universally established threshold for anaemia under 6 months, this study uses two thresholds to examine anaemia prevalence among the sample. The solid line represents the anaemia prevalence using the China Pediatrics Association threshold for infants under 6 months^(14)^. The dashed line represents the anaemia prevalence using the WHO threshold established for infants over 6 months^(3)^, which has been used in other studies of anaemia under 6 months of age

Figure 2 plots anaemia rates among the sample by month of age, using the WHO threshold (dashed line) and the China Pediatrics Association threshold (solid line). Using the WHO threshold, the prevalence of anaemia remains above 60 % under 6 months of age. Using the China Pediatrics Association threshold, the results show an anaemia prevalence of 12 % at 1 month, which increases to a prevalence of 34 % by 6 months of age. The prevalence of anaemia is lowest among infants 3 months of age (2·8 %), which corresponds to the end of the physiologic anaemia period of infancy (when fetal Hb decreases at 8–12 weeks and subsequently recovers as the infant begins to produce adult Hb)^(30)^. Aside from this natural drop in anaemia prevalence, the results show a rising trend in anaemia rates under 6 months of age.

### Factors associated with infant Hb levels

Table 3 presents the correlations between perinatal characteristics and feeding behaviours and infant Hb concentrations, controlling for socio-demographic characteristics (infant age in days, gender, birth order, maternal age and education, maternal employment status and household annual income). When examining perinatal characteristics, the data show that birth weight is significantly associated with higher Hb concentrations among both age groups. For infants under 4 months of age, an increase in birth weight by 1 kg is associated with a 4·1 g/l increase in Hb (*P* < 0·05). For infants 4–5 months of age, an increase in birth weight by 1 kg is associated with a 6·6 g/l increase in Hb (*P* < 0·01). In contrast, the results find that birth via C-section (as opposed to vaginal birth) was associated with approximately 4·6 g/l lower Hb among infants in both age groups (*P* < 0·01). We did not find significant associations with Hb levels for gestational age, whether the mother had gestational anaemia, whether the infant had illness symptoms in the past 2 weeks or whether the mother had previously experienced a miscarriage/abortion.

**Table 3:** Associations between infant characteristics and Hb concentration by age group in Sichuan Province (*n* 577)

	<4 months				4–5 months			
	*β* †	SE	*t*	95 % CI	*β*	SE	*t*	95 % CI
Birth and perinatal characteristics								
Birth weight (kg)	4·137*	2·002	2·07	0·193, 8·081	6·603**	1·862	3·55	2·937, 10·269
Delivery method (reference = vaginal birth)	−4·640**	1·599	−2·90	−7·790, −1·491	−4·578**	1·459	−3·14	−7·451, −1·706
Illness in the last 2 weeks (reference = No)	2·229	1·533	1·45	−0·791, 5·249	1·406	1·455	0·97	−1·458, 4·271
Gestational age (weeks)	−0·516	0·606	−0·85	−1·710, 0·677	−0·369	0·594	−0·62	−1·538, 0·801
Gestational anaemia (reference = no)	0·232	1·507	0·15	−2·738, 3·201	0·153	1·503	0·10	−2·806, 3·113
Mother had previous miscarriage/abortion (reference = no)	2·077	1·582	1·31	−1·039, 5·192	−0·321	1·495	−0·21	−3·264, 2·622
Feeding behaviours								
Breast-feeding status (reference = exclusive breast-feeding)								
Mixed breast-feeding	−0·597	1·565	−0·38	−3·681, 2·486	−2·979	1·926	−1·55	−6·771, 0·814
Non-breast-feeding	−1·127	4·397	−0·26	−9·789, 7·535	−10·960*	4·922	−2·23	−20·651, −1·270
Iron supplementation (reference = No)	−0·289	2·235	−0·13	−4·692, 4·113	1·037	1·779	0·58	−2·465, 4·540
Constant	109·173				98·643			
Adjusted *R* ^2^	0·2239				0·1664			
Sig. of model	0·017				0·002			
*n*	266				311			

* *P* < 0·05.

** *P* < 0·01.

† Unstandardised coefficients are listed.

Regressions control for infant age (in days), gender, birth order, maternal age (in years), maternal education level, maternal employment status and annual household income.

With regard to feeding behaviour, the results show no significant predictors for Hb concentration among infants under 4 months of age; however, for infants 4–5 months of age, not receiving any breast milk was associated with significantly lower Hb concentration. Compared with exclusively breastfed infants, non-breast-feeding infants had approximately 11·0 g/l lower Hb (*P* < 0·05). Infants that received mixed breast-feeding (i.e. fed formula or complementary foods in addition to breast milk) did not show a significant difference in Hb level compared with infants who were exclusively breastfed. Additionally, iron supplementation was not significantly associated with increase or decrease in Hb levels.

## Discussion

This study examined the prevalence of anaemia and factors associated with lower Hb among 577 infants under 6 months of age in rural China. The results found a high prevalence of anaemia, with increasing prevalence by older age in months. Lower birth weight and delivery by C-section were significantly associated with lower Hb levels among infants in both age groups (< 4 months and 4–5 months), and non-breast-feeding was significantly associated with lower Hb among infants aged 4–5 months.

The definition for anaemia is not well established under 6 months of age and differs between the WHO (under 110 g/l) and China Pediatrics Association (90 g/l for < 4 months and 100 g/l for 4–5 months). The average Hb concentration in the sample was 106·03 g/l, with 62·6 % of infants considered anaemic per the WHO definition and 20·5 % considered anaemic per the China Pediatrics Association threshold. By comparison to other world regions, prevalence of anaemia (per WHO definition) among infants under 6 months in this study is higher than that found in South Africa (50 %) and Taiwan (2·7 %) but lower than rates reported in Indonesia, Bangladesh and Peru (71, 72 and 77 %, respectively)^(10–13,31)^. The prevalence of anaemia for infants under 6 months of age per WHO definition is also similar to that of infants 6–12 months of age in rural China, which has previously been reported as between 54 and 65 %^(21,24)^. The 20·5 % prevalence we report using the China Pediatrics Association threshold for anaemia is comparable to the over 20 % rate of anaemia observed by Yang in infants under 4 months in Shaanxi, China^(15)^. Although the threshold for anaemia differs between the WHO and that China Pediatrics Association, these numbers are concerningly high per any anaemia definition and deserve further study.

Using the China Pediatrics Association thresholds, anaemia prevalence in this sample was the lowest from 1–3 months of age and then increased linearly from 3–6 months. The low anaemia prevalence at 1–3 months is most likely attributable to the ‘physiologic anaemia’ period of infancy, in which an infant’s fetal Hb levels decrease in the 8–12 weeks after birth; this triggers erythropoiesis (production of new erythrocytes by the marrow) at around 3 months, allowing for a gradual recovery in Hb levels^(30)^. Our study finds that Hb level starts to rise gradually at about 3·5 months of age as expected, returns to its original level at 4 months of age and continues to rise with increasing age, rising to nearly 30 % by 5 months. This increase in anaemia prevalence after 3 months of age is suggestive of true iron deficiency anaemia among the sample infants which is not due to physiologic causes. This increasing trend predicts a prevalence of anaemia around 40 % by 6–9 months, consistent with what others have described in rural China^(15,21)^.

The high prevalence of anaemia in rural China has important implications for overall child health and development. Anaemia is associated with higher frequency of childhood infections, as well as impaired cognitive development in childhood^(32,33)^. A study in rural China showed that 50 % of toddlers aged 2–3 years had cognitive delays and that among children 13–14 years of age the prevalence of cognitive delays was still high at 37 %^(34)^. A high prevalence of early childhood anaemia in rural China suggests that many rural children may face significant challenges to achieving their cognitive potential, in turn affecting their future earning potential and the multiple subsequent impacts thereof.

The results found that lower birth weight and delivery by C-section were significantly associated with lower Hb levels among infants under 6 months of age in rural China. The relation of birth weight to Hb is consistent with previous studies of infants over 6 months of age and the handful of studies examining infants under 6 months of age, which have consistently found a significant association of low birth weight with anaemia^(12,15,31)^. Lower birth weight infants are at high association with iron deficiency not only because they start out life with smaller iron stores but also because their faster rate of postnatal ‘catch-up’ growth (which occurs particularly in the first 6 months of life) may deplete existing iron stores^(30,35)^. For this reason, the WHO recommends providing iron supplementation to infants with birth weight under 2500 g beginning at 2 months of age^(3)^.

Delivery via C-section was also associated with significantly lower Hb among the sample. This contrasts with other previous studies, which have found no significant associations between C-section births and anaemia among infants and toddlers in China and in LMIC more generally^(23,36)^. However, one study by Li *et al.* did find C-section to be associated with anaemia at 12 months and 58 months of age^(37)^. One potential explanation for the observed association of increased anaemia amongst C-section deliveries is that infants delivered by C-section are less likely to receive delayed clamping of the umbilical cord after birth^(38)^. Early cord clamping post-delivery prevents blood from the placenta from entering the infant’s circulation, in turn decreasing both absolute number of erythrocytes and overall iron stores^(39)^. In recent years, WHO recommends a minimum 1-min delay after delivery before clamping the cord for vaginally delivered newborns to improve iron stores in the first 6 months of life^(40)^. However, the timing of cord clamping in infants delivered by C-section has not been established^(41)^. Few prevalence studies have been published on the practice of delayed cord clamping, but observational studies in both high-income and low- and middle-income settings have reported a delayed cord clamping rate of about 50 %^(42,43)^. Another possibility is that C-sections may create challenges to infant feeding. C-sections have been linked to difficulties in early initiation of breast-feeding and lower rates of excusive breast-feeding^(38,44)^. There is also some evidence that caesarean-delivered newborns may have greater difficulty with milk digestion and absorption^(45)^. All of these factors may contribute to a higher prevalence of anaemia in the caesarean-delivered group together. Given the high rate of C-section in our study (57 %), the association between C-section and low Hb in the first 6 months is concerning and points to a need for more research on hospital birth practices, infant feeding and links to anaemia.

Finally, the results showed that for infants aged 4–5 months, non-breast-feeding was associated with significantly lower Hb compared with exclusive breast-feeding, which is consistent with other studies^(15)^. This may be because, as our research team observed (and survey data showed), infants in rural areas of China are mostly fed iron-poor complementary foods, such as rice flour or porridge. Previous studies have found exclusive breast-feeding to be protective of infant iron status in areas where iron-fortified foods are not as available or are not being offered^(35,46)^. Improving caregiver knowledge about infant iron needs and appropriate complementary foods may help to improve Hb levels among non-breast-feeding infants^(47)^.

Our study makes significant contributions to the literature on infantile anaemia. At present, there are few studies of anaemia among infants under 6 months internationally, including in China. This study reveals relatively high rates of anaemia among infants under 6 months, pointing to a need for more attention on this issue. Our study also provides new evidence of associated factors for infant anaemia, which can inform public health interventions to improve early childhood nutrition and may contribute to changes in delivery practices.

We also acknowledge several limitations to this study. First, this study utilised point-of-care Hb tests and did not assess iron deficiency using other biomarkers. The use of this measure as an indicator of iron deficiency status may not be suitable in areas where the prevalence of iron deficiency is low^(48)^. However, in China, iron deficiency anaemia is the most common form of anaemia and other causes of anaemia are not prevalent, especially in rural areas^(21)^. Second, our infant feeding questionnaire relied on caregiver self-report, which may be subject to recall bias. Bias in reporting household income is similarly unavoidable. However, this feeding questionnaire is derived from the WHO *Indicators for Infant and Child Feeding* ^(29)^, and it has been used in multiple studies in China and internationally^(49,50)^. Third, our study examines a relatively small number of perinatal and feeding variables as potential risk factors, and we do not report detailed data on infant feeding or in-hospital birth experiences (e.g. the timing of cord clamping). These reasons may lead to the missing of some variables in the analyses and are possible reasons for the poor fit of the model in Table 3. In addition, a large share of the sample was ineligible^(26)^ (younger than 42 d) or refused to participate in Hb testing. Our balance test between infants who did and did not participate in Hb testing found no significant differences other than iron supplementation. Considering that infants in the group with incomplete Hb testing were fed iron supplementation significantly lower than those in the group with completed Hb testing, it may lead to a higher actual anaemia rate if all babies were tested successfully. Finally, although the study area is similar to the average level for the region in terms of economic indicators, it is possible that our sample may not be broadly representative of rural areas across China. Future studies need to disaggregate by age of month for children under 6 months while taking into account ethnic and regional differences to better understand the nature and scope of infant anaemia in rural China.

## Conclusions

This study provides reliable evidence of relatively high prevalence of anaemia among infants under 6 months in rural China than other region, with increasing prevalence of anaemia by infant age. Its prevention requires public health practitioners and policy makers should move the point of focus forward to at least 4 months of age, not just the common start at 6 months of age. Infants with low birth weight and delivered by C-section are highly associated with low Hb levels. This also requires addressing the multiple opportunities available for prevention: pregnancy, at birth and during the first 6 months of life. Future studies should further examine the link between C-section and infant anaemia, particularly in settings such as China where the prevalence of C-section is high, to develop better protocols to promote health and development in infancy.

## Supplementary material

For supplementary material accompanying this paper visit https://doi.org/10.1017/S1368980022001616. click here to view supplementary material
