# Supplementary material for: Prevalence of anaemia and associated factors among infants under 6 months in rural China
Source: Public Health Nutr. 2022 Aug 3;26(3):633–42. doi: 10.1017/S1368980022001616 (PMC9989709; doi:10.1017/S1368980022001616)
Supplement: Supplementary file 1 [file S1368980022001616sup001.pdf]

Appendix Table. Comparison of testing to non-testing of haemoglobin concentration of sample infants in rural Sichuan Province (N=728).

Variables	Hb test (n=577) (1)		No Hb test (n=151) (2)		Difference (1) – (2)
	n or mean	% or SD	n or mean	% or SD	P value
Demographic and Socioeconomic Characteristics					
Age (in week)	3.71	1.60	3.64	1.68	0.469
Gender					0.258
Male	323	81.77	72	18.23	
Female	254	78.40	70	21.60	
Birth order					0.422
First	249	81.64	56	18.36	
Second or higher	328	79.23	86	20.77	
Maternal age (in year)	28.44	6.16	27.74	4.18	0.202
Maternal education level					0.258
≤ 9 years	230	78.23	64	21.77	
＞9 years	347	81.65	78	18.35	
Maternal is currently employed					0.355
No	438	79.49	113	20.51	
Yes	139	82.74	29	17.26	
Annual household income (¥, 10^4)	5	7	6	6.4	0.626
Birth and Perinatal Characteristics					
Gestational age (in week)	38.83	1.47	38.97	1.43	0.151
Birth weight (in kilogram)	3.23	0.45	3.24	4.47	0.785
Delivery method					0.515
Natural vaginal birth	249	81.37	57	18.63	
Cesarean sections	328	79.42	85	20.58	
Gestational complication					0.636
No	306	80.53	74	19.47	
Yes	271	79.01	72	20.99	
Mother's history of miscarriage/abortion					0.289
No	310	78.28	86	21.72	
Yes	239	81.57	54	18.43	
Feeding Behavior Related Characteristics					
Breastfeeding status					0.894
Exclusive breastfeeding	177	79.37	46	20.63	
Non-exclusive breastfeeding	372	80.52	90	19.48	
Non-breastfeeding	28	82.35	6	17.65	
Iron supplemented					0.036
No	474	78.87	127	21.13	
Yes	103	87.29	15	12.71	
